# Cardiorespiratory Functioning in Youth with Persistent Post-Concussion Symptoms: A Pilot Study

**DOI:** 10.3390/jcm10040561

**Published:** 2021-02-03

**Authors:** Aliyah Snyder, Christopher Sheridan, Alexandra Tanner, Kevin Bickart, Molly Sullan, Michelle Craske, Meeryo Choe, Talin Babikian, Christopher Giza, Robert Asarnow

**Affiliations:** 1 Department of Psychiatry, University of California, Los Angeles, CA 90095, USA; tbabikian@mednet.ucla.edu (T.B.); rasarnow@mednet.ucla.edu (R.A.); 2 UCLA Steve Tisch BrainSPORT Program, Los Angeles, CA 90095, USA; casherid@wakehealth.edu (C.S.); kbickart@mednet.ucla.edu (K.B.); mchoe@mednet.ucla.edu (M.C.); cgiza@mednet.ucla.edu (C.G.); 3 Department of Radiology, Wake Forest School of Medicine, Winston-Salem, NC 27101, USA; 4 Department of Psychology, University of California, Los Angeles, CA 90095, USA; tannera@ucla.edu (A.T.); craske@psych.ucla.edu (M.C.); 5 Departments of Neurology and Neuropsychiatry, David Geffen School of Medicine at UCLA, Los Angeles, CA 90095, USA; 6 Department of Psychiatry, Psychology Service, University of California, San Diego, CA 92093, USA; msullan@health.ucsd.edu; 7 VA San Diego Healthcare System, San Diego, CA 92161, USA; 8 UCLA Mattel Children’s Hospital, Los Angeles, CA 90095, USA; 9 Department of Pediatrics, David Geffen School of Medicine at UCLA, Los Angeles, CA 90095, USA; 10 Department of Neurosurgery, David Geffen School of Medicine at UCLA, Los Angeles, CA 90095, USA

**Keywords:** autonomic functioning, concussion, cardiorespiratory, brain injury, end-tidal CO_2_, post-concussion symptoms, mild traumatic brain injury

## Abstract

Dysregulation of the autonomic nervous system (ANS) may play an important role in the development and maintenance of persistent post-concussive symptoms (PPCS). Post-injury breathing dysfunction, which is influenced by the ANS, has not been well-studied in youth. This study evaluated cardiorespiratory functioning at baseline in youth patients with PPCS and examined the relationship of cardiorespiratory variables with neurobehavioral outcomes. Participants were between the ages of 13–25 in two groups: (1) Patients with PPCS (concussion within the past 2–16 months; *n* = 13) and (2) non-injured controls (*n* = 12). Capnometry was used to obtain end-tidal CO_2_ (EtCO_2_), oxygen saturation (SaO_2_), respiration rate (RR), and pulse rate (PR) at seated rest. PPCS participants exhibited a reduced mean value of EtCO_2_ in exhaled breath (M = 36.3 mmHg, SD = 2.86 mmHg) and an altered inter-correlation between EtCO_2_ and RR compared to controls. Neurobehavioral outcomes including depression, severity of self-reported concussion symptoms, cognitive catastrophizing, and psychomotor processing speed were correlated with cardiorespiratory variables when the groups were combined. Overall, results from this study suggest that breathing dynamics may be altered in youth with PPCS and that cardiorespiratory outcomes could be related to a dimension of neurobehavioral outcomes associated with poorer recovery from concussion.

## 1. Introduction

Persistent post-concussion symptoms (PPCS), defined by most as symptoms lasting longer than 2 to 3 months post-injury, affect a significant minority of patients who sustain a concussion [1,2,3,4,5,6]. Multimodal rehabilitation models for PPCS may be beneficial, particularly those that include interventions such as exercise, physiotherapy, and/or cognitive behavioral treatment [7,8,9,10,11]. However, the specific interventions that render multimodal treatments successful in patients with PPCS are either difficult to isolate or have yet to be fully elucidated. Although biopsychosocial frameworks and other studies describe personal, injury, and environmental factors that interact with recovery [12,13,14,15], a cohesive model for the mechanisms of PPCS has not yet been established. 

Post-concussion symptoms are not specific to concussion and also occur in the general population [16,17]. The role of the autonomic nervous system (ANS) in the development and maintenance of post-concussion symptoms has emerged as a potentially important component of PPCS etiological frameworks [18,19,20]. The ANS is responsible for maintenance of internal homeostasis through the regulating actions of its two branches, the sympathetic nervous system (SNS) and parasympathetic nervous system (PNS). The ANS is involved in conscious and unconscious regulation of critical bodily responses including changes in heart rate, blood pressure, breathing dynamics, gastrointestinal response, oculomotor adjustment, and thermoregulation, among others. There is strong overlap between symptoms associated with dysregulated ANS responses in other medical conditions (e.g., chronic pain, migraine, lupus) and those most commonly reported in concussion patients (e.g., headache, fatigue, dizziness, brain fog) [21,22,23,24,25]. Brain regions and networks critical to the central control of ANS branches, specifically the dynamic responses of the SNS and PNS, are highly vulnerable to the biomechanical forces of brain injury, even on the milder end of the severity spectrum [26,27,28]. In a study of patients with PPCS aged 13–18 years, autonomic dysfunction was identified in 70.6% of the 36 participants tested for orthostatic intolerance within 6 months post-injury [29]. Other evidence of impaired autonomic regulation of cardiovascular responses has been reliably detected in patients during acute and chronic phases of injury, primarily through measurement of heart rate variability [30,31,32,33,34].

Cardiorespiratory functioning, especially breathing dynamics, can also provide insight into ANS functioning and may be particularly applicable to the study of concussion and PPCS. Capnometry is a relatively rapid and non-invasive tool that permits the measurement of carbon dioxide concentration in exhaled breath. End-tidal CO_2_ (EtCO_2_), the CO_2_ concentration obtained at the end of an expiratory phase, has been linked to several neurobehavioral processes relevant to concussion recovery. In particular, the presence of hypocapnia (lower than normal levels of EtCO_2_) is commonly reported following or sometimes preceding panic attacks [35,36,37]. Periods of hyperventilation, leading to hypocapnia, can result in acute respiratory alkalosis and symptoms such as dizziness, light-headedness, confusion, and syncope [38,39,40], which are also common to concussion. Differences in EtCO_2_ concentrations at baseline can be detected in patients with a variety of medical conditions including migraine and chronic pain, suggesting ongoing alterations in autonomic regulation. In individuals with chronic anxiety, difficulties with breathing are often reported and can take the form of pathological breathlessness or hyperventilation [37,41,42].

The relationship between cardiorespiratory function and symptom experience following concussion is understudied. At present, one study demonstrated that adult patients may exhibit altered EtCO_2_ at baseline compared to controls [43], but more evidence is needed to determine whether changes in ANS dysfunction are associated with the etiology of PPCS, especially in youth patients with PPCS. As such, the purpose of this pilot study was to examine cardiorespiratory functioning, as measured by capnometry, in youth patients with PPCS and its relationship with neurobehavioral outcomes (e.g., neurocognitive functioning, mood, sleep) compared to non-injured controls. 

## 2. Experimental Section

### 2.1. Study Design and Setting

Data were obtained from the pre-intervention baseline of a prospective case-control study. The goal of the trial was to examine the effect of psychological intervention on youth patients with persistent post-concussion symptoms. Participants included concussion patients and age-matched non-injured controls recruited from the community. Data collection occurred between June 2018 and February 2020. Approval was secured from the appropriate Institutional Review Board and informed consent and assent (if under the age of 18) were obtained for all participants.

### 2.2. Participants

Participants in the concussion group were recruited from university-affiliated concussion clinics in Los Angeles, CA, USA and non-injured control participants were recruited from the local community setting, chiefly through a university. Eligibility criteria for the concussion group were as follows: between 13–25 years of age at the time of assessment, history of concussion diagnosed by a medical provider, within 2 to 16 months post-injury at the time of assessment, and experiencing persistent concussion symptoms along with evidence of behavioral avoidance due to symptoms (i.e., avoiding full participation in typical, pre-injury activities for fear of symptom exacerbation). Behavioral avoidance was assessed through a screening measure developed for this project, called the Return to Activity Avoidance Inventory (RAAvI) (see Supplementary Materials, Figure S1). The RAAvI measure required participants to identify three activities they were actively avoiding due to concern about symptom provocation or exacerbation. Participants then rated their level of avoidance on a Likert scale (0 to 6 where 0 = likely to avoid the activity none of the time and 6 = likely to avoid the activity all of the time) and their perceived level of emotional and/or physical distress if they were to participate in the activity, also on a Likert scale (0 to 6 where 0 = likely to experience no physical or emotional distress and 6 = likely to experience extreme physical or emotional distress). Participants were selected if they were able to identify at least one activity that they actively avoided (1 or higher on Likert rating).

Participants were excluded if they had any comorbid neurological conditions (including history of stroke, seizure disorder, moderate to severe traumatic brain injury, anoxia), severe cardiovascular conditions, history of psychosis, current substance abuse or dependence, severe symptoms of depression and/or anxiety at the time of the assessment (as determined by self-report questionnaires), or were unable to speak English. Participants who were prescribed psychotropic medication for treatment of concussion-related symptoms (e.g., headache and migraine) were permitted to be enrolled in the study as long as they were not prescribed serotonin-norepinephrine reuptake inhibitors (e.g., desvenlafaxine, venlafaxine, duloxetine, etc.) given their impact on autonomic function. Control participants were eligible for the study if they were able to speak English and were between 13–25 years old. They were excluded if they had a history of concussion within the past 12 months or were experiencing persistent post-concussive symptoms from a previous injury. The neurological and psychiatric exclusion criteria described above were also applied to controls, but there were no restrictions on current medications.

### 2.3. Instrumentation and Outcome Measures

General demographic and background information was obtained via self-report questionnaire and included age, sex, education, as well as self and family medical/psychiatric history. Neurobehavioral outcomes were acquired via standardized self-report questionnaires and neurocognitive performance was obtained through paper-and-pencil testing administered by trained research staff. All data were collected during a single session and assessments were administered in a fixed order.

#### 2.3.1. Neurobehavioral Measures

Standardized self-report measures were administered to evaluate post-concussion neurobehavioral function, including symptom burden, mood, sleep quality, and pain catastrophizing. The adolescent form (ages 13–18) of the Post-Concussion Symptom Inventory (PCSI-SR13; [44]) was used, which is a 21-item graded checklist. All participants were asked to rate their current symptoms (covering the day of and the day before the assessment). Symptom of depression and anxiety were evaluated using the raw scores from the Beck Depression Inventory, 2nd Edition (BDI-II; [45]) and the Beck Anxiety Inventory (BAI; [46]). The BDI-II is a 21-item scale that asks participants to evaluate how they have been feeling over the past two weeks. Similarly, the BAI is a 21-item graded scale that asks participants to rate how much they have been bothered by anxiety symptoms over the past week. Higher scores on both the BDI-II and BAI suggest greater symptoms of depression and anxiety, respectively. The global composite score on the Pittsburgh Sleep Quality Index (PSQI; [47]) was used to characterize sleep quality. Participants answered based on their sleep experience over the past month, and the global composite was calculated using the 7 components scores of sleep quality (i.e., subjective sleep quality, sleep latency, sleep duration, sleep efficiency, sleep disturbance, use of sleep medication, and daytime dysfunction), with higher scores indicating worse sleep quality. The Pain Catastrophizing Scale (PCS; [48]) is a 13-item measure of cognitive catastrophizing that is associated with the perception and experience of pain. Higher raw scores on the PCS indicate a more maladaptive cognitive coping style in response to pain. Participants were administered an abbreviated battery of the Repeatable Battery for the Assessment of Neuropsychological Status (RBANS; [49]). Subtests were selected to focus on cognitive domains typically affected by concussion, including attention (Digit Span and Coding), learning and memory (List Learning and List Recall), and verbal fluency (Semantic Fluency).

#### 2.3.2. Capnometry Instrumentation and Assessment

The Capnostream^TM^ 35 portable capnometer (Medtronic Inc., Minneapolis, MN, USA) was used to measure the primary outcome of interest, EtCO_2_, as well as other cardiorespiratory metrics including pulse rate (PR, number of heart beats per minute), respiration rate (RR, number of breaths taken per minute), and oxygen saturation (SaO2, percentage of oxygen bound to hemoglobin). EtCO_2_ and RR were measured via nasal cannula and PR and SaO_2_ were collected via pulse oximeter, both of which were connected to the device. Capnometry allows for non-invasive, real-time monitoring of cardiorespiratory outcomes and data were sampled at a rate of f_s_ = 1. Data were obtained after the neurobehavioral assessments were administered and a brief (5–10 min) break was provided to participants. Cardiorespiratory values were averaged over a 5-min continuous recording period during which participants remained in an upright, seated position and were instructed to breathe normally and avoid talking. Capnometry units were initially calibrated prior to the study and no re-calibration took place during data collection in accordance with manufacturer’s instructions.

#### 2.3.3. Statistical Analyses

Data were inspected for normality and outlier cardiorespiratory responses were removed only if likely to be discrepant, non-biologic values (i.e., 0). Two participants were excluded (one in each group) due to nasal cannula dysfunction. Demographic comparisons between groups were analyzed via chi-square analyses, Fisher’s exact test, and parametric *t*-tests. Statistical procedures included parametric (independent samples) and non-parametric (Mann-Whitney) *t*-tests to compare groups on cardiorespiratory, demographic, and neurobehavioral variables (i.e., EtCO_2_, PR, RR). Separate one-way analyses of covariance (ANCOVA) were included to evaluate potential confounding effects of age and sex on cardiorespiratory variables. Pearson bi-variate and Spearman rank order correlations were used to examine the relationships between variables, and correlation coefficients were compared using Fisher’s z transformation. Data were analyzed using IBM SPSS Statistics (IBM Corp., Armonk, NY, USA), version 26.0 [50].

## 3. Results

A total of 13 PPCS participants and 12 controls were recruited from UCLA outpatient concussion clinics and surrounding community. Participant demographics are presented in Table 1. Overall, the two groups were broadly similar when sex-distribution and medical history were compared. The PPCS group was two years younger on average, *t*(23) = 2.31, *p* = 0.03. Additionally, all participants in the PPCS group reported having sustained at least one previous concussion, whereas no participant in the control group reported prior history of concussion, *t*(23) = −6.40, *p* < 0.001. 

The PPCS group demonstrated a significantly higher current neurobehavioral burden than the control group (see Table 2). The PPCS group exhibited higher pain catastrophizing scores and higher self-reported symptoms. On both the PCSI and PSQI, the PPCS group reported more sleep problems, but the groups differed significantly only on the PCSI sleep subscale. Although not statistically significant, the PPCS group trended toward a greater emotional burden on the BDI-II and the emotional subscale of the PSCI. Although PPCS patients were selected because they exhibited activity avoidance due to symptoms, they did not report clinically or statistically higher levels of anxiety symptoms. Along with differences on the self-report measures, the PPCS group demonstrated significantly poorer neurocognitive performance on subtests of the RBANS measuring learning and attention (List Learning Total Score), semantic verbal fluency (Semantic Fluency), and verbal recall (List Recall). Effect sizes for these differences were large (Cohen’s *d* range, 0.80–1.43).

### 3.1. Cardiorespiratory Variables by Group

**Figure 1:**
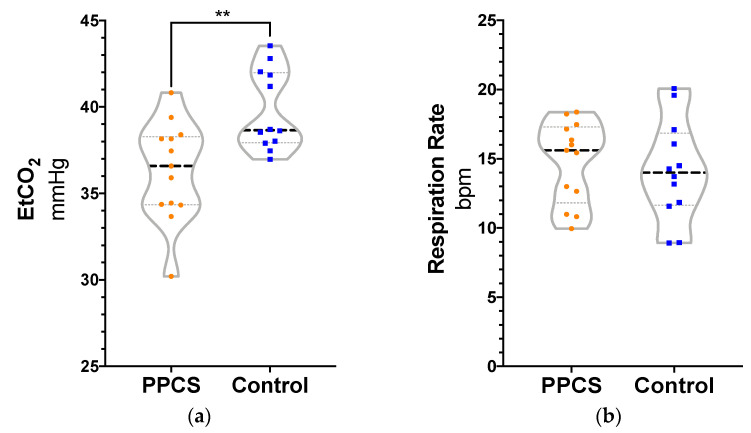

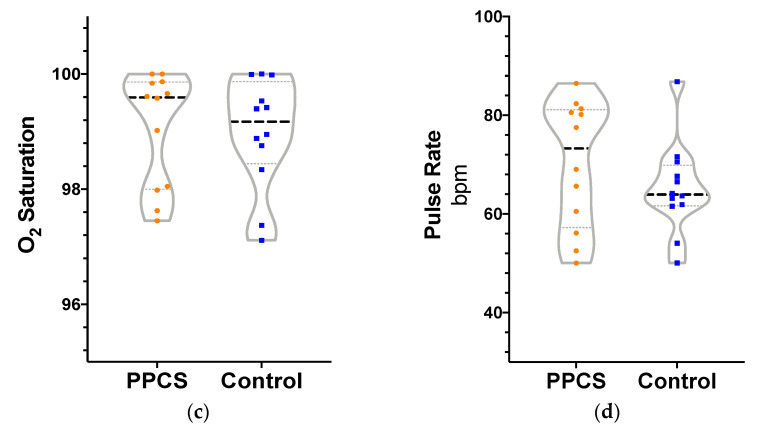
Truncated violin graphs for cardiorespiratory variables by group. (**a**) EtCO_2_ (**b**) Respiration rate (**c**) Oxygen saturation (**d**) Pulse rate. The lines represent quartile ranges (gray) and median values (black). bpm = beats per minute (respiration rate) or breaths per minute (pulse rate).

Independent samples *t*-test revealed significantly lower EtCO_2_ in PPCS patients (*M* = 36.30, *SD* = 2.86) compared to controls (*M* = 39.80, *SD* = 2.30), *t*(23) = 3.36, *p* = 0.003, *d* = 1.35. There were no significant statistical differences between groups for RR, PR, or SaO_2_, although there was greater variability in the pulse rate of the PPCS group (see Figure 1). Separate one-way ANCOVAs were employed with age as a covariate given that age was significantly different between groups. Quade’s rank analysis of covariance was used for SaO_2_. There were no statistically significant main effects of age on the cardiorespiratory variables, with the exception of RR, *F*(1, 24) = 11.86, *p* = 0.002, η^2^ = 0.35. Older age was associated with lower RR (b = −0.77). The inclusion of age as a covariate did not change the pattern of significant findings by group reported above. In separate ANCOVAs, sex was not a significant covariate for any of the cardiorespiratory variables. For the PPCS group, bi-variate correlations of days since injury and cardiorespiratory outcomes produced no statistically significant relationships.

When the relationships between EtCO_2_ and the other cardiorespiratory variables were examined, the control group demonstrated a strong, negative correlation between EtCO_2_ and SaO_2_ using Spearman’s rank-order correlation, *r*(22) = −0.71, *p* = 0.009. However, for the PPCS group, there were no significant intercorrelations between EtCO_2_ and the other cardiorespiratory variables, including SaO_2_, *r*(22) = −0.20, *p* = −0.54. 

The individual inter-correlations between EtCO_2,_ SaO_2_, PR, and RR were computed and then compared by group. Results demonstrated that the intercorrelation between EtCO_2_ and RR were significantly different between groups (independent samples *t*-test), *t*(23) = −2.22, *p* = 0.04, such that the control group participants were more likely to exhibit a negative relationship (*M* = −0.25, *SD* = 0.28) than participants in the PPCS group (*M* = 0.01, *SD* = 0.30). No other inter-correlation values were significantly different between the groups. 

### 3.2. Cardiorespiratory Variables and Neurobehavioral Outcomes

The relationships between cardiorespiratory variables and neurobehavioral outcomes were also examined via Pearson and Spearman rank-order correlations (see Table 3). Correlations were examined both by group and by the overall combined sample to better assess the dimensionality of cardiorespiratory and neurobehavioral variables and to provide estimates of effect. When the groups were combined, EtCO_2_ was significantly associated with pain catastrophizing, self-reported symptoms, and depression; lower EtCO_2_ was related to greater symptom burden on these scales. For SaO_2_, total self-reported PCSI symptoms and depression were strongly correlated suggesting higher baseline SaO_2_ is associated with greater symptom burden, but only in the PPCS group. RR was positively correlated with age, depression, and psychomotor processing speed performance (i.e., Coding) when the groups were combined. Lastly, PR was positively correlated with pain catastrophizing, anxiety, depression, and psychomotor processing speed when the groups were combined. Although there was trending variation in the pattern of correlations between cardiorespiratory and demographic/neurobehavioral outcomes when the groups were examined independently, these differences were not statistically different by group when correlation coefficients were compared using Fisher’s z transformation. 

## 4. Discussion

The results of this pilot study indicate that baseline cardiorespiratory response may be altered in youth patients with PPCS who exhibit behavioral avoidance compared to control participants. At baseline, PPCS participants exhibited a significantly reduced mean value of EtCO_2_ in exhaled breath (*M* = 36.3 mmHg, *SD* = 2.86 mmHg), falling on the lower end of the typical ranges (38.7 ± 2.7 mmHg) identified by a large study of ventilatory characteristics of young, healthy adults [51]. Other studies identify 35 mmHg as a clinical cut point of EtCO_2_ for hypocapnia in emergency or surgical settings [52,53], but EtCO_2_ values below 37 mmHg have also been used as evidence of mild hypocapnia [54,55]. Taken together, results from this study suggest the possibility of a mild degree of breathing dysregulation in our sample (62% of PPCS vs. 8% of Controls with EtCO_2_ < 37 mmHg). 

Previous literature has shown that imbalances in arterial carbon dioxide tension leading to hypocapnia are generally due to hyperventilation, an umbrella term that includes deep and/or rapid breathing. In our study, the number of breaths per minute (RR) was not different between the groups, suggesting that the decrease in EtCO_2_ observed in the PPCS patients is not simply attributable to breathing faster at baseline. Instead, the relationship between RR and EtCO_2_ in PPCS patients is different from controls, potentially indicating that another mechanism such as tidal volume (the depth or amount of air inhaled during a normal breath), which was not measured by this study, may be responsible for the lower EtCO_2_ in PPCS patients. Given the small sample size, there are other factors that may have influenced these results, including medication effects, environmental exposure to smoke or pollutants, and timing and breath holding, among others that were not measured. Future research is needed to confirm these results and assess other factors that may mediate or moderate the relationship between PPCS and EtCO_2_.

Our findings of possible mild hypocapnia in PPCS patients conflicts with findings from Siedlecki et al. of mild hypercapnia in PPCS patients. The discrepancy between the two studies may be accounted for by methodological differences in sample characteristics and measurement parameters. Siedlecki et al.’s study reported on a smaller sample of slightly older PPCS patients (16–29 years old; *n* = 5). Additionally, the present study reported average EtCO_2_ over a 5-min baseline assessment compared to baseline data obtained for 20 s in Siedlecki et al.’s work. Lastly, our study specifically selected for PPCS patients who presented with an activity avoidance component, which increased the likelihood that they might exhibit cardiorespiratory effects of increased arousal and anxiety.

Typical cardiovascular reactions to stress are characterized by cardiovascular sympathetic responses (i.e., increased heart rate, tidal volume, and breath frequency) and a reciprocal decrease in parasympathetic tone to help the body meet the resource demands of an internal or external challenge [56]. For patients with autonomic dysregulation, especially those with an anxiety disorder, chronic arousal alters breathing dynamics both at rest and in response to stress [57,58]. Breathing dysregulation is generally accompanied by corresponding increase in ANS-mediated physical, cognitive, and mood symptoms (e.g., headache, balance problems, dizziness, intrusive thoughts, panic, etc.). Consistent with this literature, the present study demonstrated an association between certain neurobehavioral outcomes, namely depression symptoms, self-reported concussion symptoms, cognitive catastrophizing, and slower psychomotor processing speed, and cardiorespiratory variables when the groups were examined together. Neurobehavioral measures are dimensional, and combining groups effectively increases the variance and may account for the stronger magnitude of correlations when the groups were examined together. Lower EtCO_2_ and higher PR at rest appeared to be most strongly and consistently related to worse neurobehavioral outcomes in our sample. The groups did not statistically differ in their relationships between the cardiorespiratory variables and neurobehavioral outcomes. Our findings suggest that cardiorespiratory function overall could be related to neurobehavioral outcomes associated with poor recovery from concussion. Moreover, our findings overlap with symptom and physiological evidence of autonomic dysregulation in other medical, psychiatric, and research populations.

Self-reported anxiety was not strongly related to the cardiorespiratory outcomes measured in this study, but pain catastrophizing, a type of negative cognitive response to anticipated or experienced pain, was correlated. Another study examining transcutaneous partial pressure of arterial carbon dioxide in patients with chronic neck pain also found that pain catastrophizing was more strongly related to changes in respiration than self-reported anxiety [59]. Many studies have demonstrated a link between EtCO_2_ and anxiety, particularly panic disorders [55,57,60]. Subjects who experienced a high frequency of internalizing or self-directed thoughts also tended to exhibit decreased EtCO_2_ [61]. Interestingly, female youth and adolescents with conduct disorder (an externalizing disorder) did not exhibit anomalies in the autonomic nervous system unless they were also diagnosed with a co-morbid internalizing disorder, in which case there was evidence of reduced parasympathetic activity in cardiovascular responses [62]. In concussion, several psychological factors including pain catastrophizing, cogniphobia (fear of cognitive exertion due to headache), and fear avoidance interfere with recovery from injury [63,64]. Pain catastrophizing in particular mediates the relationship between anxiety and post-concussion symptoms [65]. The present study is the first to demonstrate a possible link between pain catastrophizing and changes in cardiorespiratory function in PPCS patients.

Potential mechanisms or models for how PPCS could lead to altered cardiorespiratory patterns at baseline are most effectively viewed through the neurobiopsychosocial lens, a complex interaction of pre-morbid and post-injury biological, psychiatric, personality, and environmental factors. The pathophysiology of acute concussion is known to impact central ANS control resulting in autonomic abnormalities affecting cardiovascular and cerebrovascular function that may remain far into the chronic post-injury period for some patients and contribute to persistent symptomatology [27,30,66,67,68]. Within a few days post-injury, patients with mild traumatic brain injury have demonstrated impaired ability to modulate cerebral blood flow (i.e., cerebral vasoreactivity) in response to CO_2_ changes from respiratory challenge [68]. Greater impairment in cerebral vasoreactivity has also been associated with increased post-concussive symptom burden [69], highlighting the potential reciprocal impact of altered CO_2_ dynamics on recovery after brain injury. Heart rate variability (HRV), an index of autonomic function that is more robustly studied in patients with concussion, is associated with symptom burden and concussion recovery [70]. HRV is closely linked to breathing, with as much as 70% of the variation in HRV accounted for by respiratory dynamics [71,72]. Pöyhönen et al. [72] identified independent contributions of EtCO_2_ and respiration rate to the high-frequency power spectrum in HRV, which is an index of vagal activity. Taken together, this study provides additional methodological perspective and data for examining cardiorespiratory function as an indicator of autonomic functioning in patients with PPCS.

Imbalance in the responsivity of sympathetic and, especially, parasympathetic cardiovascular responses may lead to a pattern of dysregulated autonomic adaptation to stress, which is then reinforced through avoidance for some patients. In concussion clinics, patients often report behavioral avoidance (i.e., avoiding or reducing participation in social, physical, or cognitive activities) as a way to manage concussion-induced symptoms (sometimes encouraged by their well-meaning treating clinicians). However, this approach is now known to be a double-edged sword and can drive persistent symptoms instead. Said another way, ANS dysfunction initiated by concussion may become self-propagating for some patients with avoidant coping styles whereby the feedback between ANS dysfunction and neuropsychological perturbations may drive further or more persistent ANS dysfunction. The results of this study highlight cardiorespiratory function as a potential target for future investigation to better understand its role in post-injury autonomic regulation. Indeed, hypocapnia on its own has the ability to prolong and initiate systemic pathology by impacting cellular metabolism, blood oxygenation, and neurotransmitter systems [73]. Dysregulated breathing can also trigger anxiety, particularly panic symptoms, in otherwise healthy participants [74].

Additional research is needed on cardiorespiratory dynamics following concussion, particularly to uncover the specific differences in respiration following PPCS, especially with respect to tidal volume. Our study was not designed to interrogate a causal relationship between breathing dynamics and neurobehavioral functioning post-concussion, and it is likely that no straightforward relationship exists given how tightly coupled and reciprocally interactive they are. It is plausible that the PPCS group may have experienced lower EtCO_2_ prior to their injury, since reduced EtCO_2_ has also been linked to personality differences [60], specifically on the neuroticism scale of the NEO Personality Inventory, (the tendency to worry and experience negative emotions) [75]. Negative affectivity is associated with greater interoceptive attention and negative interpretation of physiological sensations [76], both of which could predispose individuals with this personality characteristic to more problematic concussion recoveries. Sex differences were not found in this study likely due to limited sample size, but previous work has identified slightly higher resting EtCO_2_ in men compared to women [75]. Given the discrepancies in concussion symptom reporting between sexes, future studies should examine sex differences in breathing following injury. 

Hypocapnia is a treatable phenomenon and future efforts might examine the impact of experimentally altering breathing to determine its more precise impacts on neurobehavioral outcomes after concussion. EtCO_2_ can also be used to index physiological response to a stressor or intervention [51]. Controlled breathing practices such as capnometry-assisted respiratory training (CART) have demonstrated efficacy in improving breathing dysregulation [77], autonomic modulation [78], pain responsivity [79]. CART can be easily combined with cognitive-behavioral treatment or psychoeducation interventions following concussion. Physical exercise is also a common intervention target for autonomic disruption in other conditions such as postural orthostatic tachycardia syndrome (POTS) and approaches that combine controlled breathing and physical exercise, may be especially effective in increasing autonomic control [80,81,82].

Several limitations should be acknowledged in this work. Firstly, the generalizability of these findings is tempered by the small sample size and evidence of heterogeneity within the sample. Findings should be expanded and replicated before they are used to guide clinical practice as the clinical utility of these results is yet to be seen. Additionally, the sample specifically selected individuals with evidence of behavioral avoidance; the cardiorespiratory findings may not extend to patients with PPCS who do not report avoidant behavior or to older adults given that EtCO_2_ may impacted by age. Other demographic factors, such as socioeconomic status, family and social environment, community setting, race, and ethnicity, were not collected as part of this study, but should be included in future efforts to describe ANS functioning post-concussion, because ANS function can be impacted by environmental and social determinants of health that may interact with the results presented [83,84]. Lastly, the present study utilized correlational relationships, which cannot offer evidence of directionality or causation between measures. In future studies, additional metrics such as tidal or minute volume are important to collect alongside EtCO_2_, RR, and PR to better understand the etiology of hypocapnia in PPCS patients. Longitudinal assessment and experimental study designs that include paced breathing and/or controlled CO_2_ manipulation would also produce more robust information about breathing dynamics and their impact on longer-term outcomes in this population.

## Figures and Tables

**Table 1:** Demographic information and injury characteristics by group.

	PPCS	Control	*p*-Value
Demographics	(*n* = 13)	(*n* = 12)	
Sex (*n*)			
Male (*n*)	4	6	0.33
Female (*n*)	9	6	
Age (*years*)	16.15 (1.86)	18.50 (3.12)	0.03 *
Education completed (*years*)	9.77 (2.01)	12.58 (2.97)	0.01 *
Medical History			
Migraine (*yes*)	3	2	1
Depression (*yes*)	1	1	1
Anxiety (*yes*)	5	1	0.16
AD/HD (*yes*)	1	1	1
Learning disorder (*yes*)	1	0	1
Taking medication (*yes*)	4	2	0.64
Previously diagnosed concussions	2.00 (1.08)	0 (0.0)	<0.001 **
Days since most recent injury	138.85 (75.76)	--	--

Note: Values are presented in mean (SD) form unless noted otherwise. * *p*-value < 0.05; ** *p*-value < 0.01. AD/HD = Attention-Deficit/ Hyperactivity Disorder.

**Table 2:** Neurobehavioral outcomes by group.

	PPCS	Control	*p*-Value
Neurobehavioral Outcomes	(*n* = 13)	(*n* = 12)	
Pain Catastrophizing Scale	25.15 (9.66)	12.33 (8.25)	0.002 **
PCSI			
*Physical*	12.08 (8.77)	3.42 (5.25)	0.008 **
*Emotional*	8.62 (5.84)	4.67 (5.84)	.05
*Cognitive*	12.85 (8.03)	3.58 (5.48)	0.004 **
*Sleep/Fatigue*	5.31 (3.99)	2.08 (2.35)	0.03 *
*Total*	39.38 (23.68)	13.75 (16.86)	0.002 **
*Pre-Injury Total*	5.38 (6.80)	--	--
Beck Anxiety Inventory	10.25 (7.57)	7.58 (6.76)	0.35
Beck Depression Inventory, 2nd Ed.	14.08 (7.32)	7.67 (8.60)	0.06
Pittsburgh Sleep Quality Index	8.58 (3.66)	6.33 (3.92)	0.16
Neurocognitive Outcomes			
RBANS			
*List Learning Total Score*	27.38 (4.23)	32.17 (2.33)	0.002 **
*Semantic Fluency*	17.92 (2.87)	21.92 (3.34)	0.004 **
*Coding*	52.85 (8.00)	59.33 (9.08)	0.07
*List Recall*	6.38 (1.81)	8.42 (0.90)	0.002 **

Note: Values are presented in mean (SD) form unless noted otherwise. * *p*-value < 0.05; ** *p*-value < 0.01; PCSI = Post-Concussion Symptom Inventory, RBANS = Repeatable Battery for the Assessment of Neuropsychological Status.

**Table 3:** Correlation coefficients of neurobehavioral outcomes and cardiorespiratory variables.

	EtCO_2_ (mmHg)			SaO_2_ (%)			RR (Breaths per Minute)			PR (Beats per Minute)		
	PPCS	Control	Combined	PPCS	Control	Combined	PPCS	Control	Combined	PPCS	Control	Combined
Neurobehavioral Outcomes	(*n* = 13)	(*n* = 12)		(*n* = 12)	(*n* = 12)		(*n* = 13)	(*n* = 12)		(*n* = 13)	(*n* = 12)	
Pain Catastrophizing Scale	−0.09	−0.16	−0.42 *	0.34	0.02	0.15	−0.07	0.5	0.22	0.38	0.58 *	0.50 *
*Rumination*	0.03	−0.22	−0.40	0.07	−0.18	0.01	0.00	0.52	0.27	0.38	0.62 *	0.51 *
*Magnification*	−0.12	0.03	−0.21	0.5	−0.04	0.24	−0.29	0.34	0.01	0.16	0.33	0.26
*Helplessness*	−0.14	−0.13	−0.45 *	0.38	0.13	0.2	0.04	0.37	0.23	0.42	0.45	.47 *
*Total*	−0.09	−0.16	−0.42 *	0.34	0.02	0.15	−0.07	0.5	0.22	0.38	0.58 *	0.50 *
PCSI												
*Physical*	−0.07	−0.34	−0.42 *	0.43	0.26	0.35	0.05	−0.22	−0.02	−0.07	−0.02	0.06
*Emotional*	−0.35	−0.22	−0.42 *	0.69 *	0.18	0.38	−0.02	0.31	0.18	−0.01	0.54	0.27
*Cognitive*	−0.20	−0.16	−0.45 *	0.58 *	0.14	0.38	0.20	0.10	0.18	−0.17	0.23	0.09
*Sleep/Fatigue*	−0.05	−0.43	−0.38	0.29	−0.18	0.20	0.25	0.14	0.22	−0.28	0.43	0.03
*Total*	−0.17	−0.26	−0.46 *	0.70 *	0.07	0.38	0.15	0.09	0.15	−0.12	0.32	0.14
*Pre-Injury Total*	0.26	--	--	0.24	--	--	−0.13	--	--	−0.24	--	--
Beck Anxiety Inventory	−0.31	−0.18	−0.32	0.46	−0.03	0.16	−0.12	0.22	0.08	0.47	0.43	0.47 *
Beck Depression Inventory, 2nd Ed.	−0.53	−0.35	−0.55 **	0.73 *	0.16	0.35	0.37	0.42	0.41 *	0.67 *	0.53	0.61 **
Pittsburgh Sleep Quality Index	0.10	0.31	−0.01	0.42	−0.09	0.08	0.36	0.29	0.32	−0.19	0.41	0.14
Neurocognitive Outcomes												
RBANS												
*List Learning Total Score*	0.05	0.13	0.38	0.03	−0.03	−0.02	−0.34	0.05	−0.20	−0.16	0.32	−0.16
*Semantic Fluency*	−0.12	0.28	0.36	−0.07	−0.51	−0.32	0.38	−0.42	−0.12	0.18	−0.51	−0.23
*Coding*	−0.15	0.33	0.26	−0.41	−0.01	−0.20	−0.33	−0.60 *	−0.48 *	−0.15	−0.65 *	−0.42 *
*List Recall*	0.05	0.03	0.37	−0.18	0.15	−0.16	−0.1	−0.38	−0.21	0.23	−0.04	−0.02

Note: Value are correlation coefficients (*R*). * *p* < 0.05, ** *p* < 0.01; EtCO_2_ = End-tidal carbon dioxide, SaO_2_ = Percent oxygen saturation, PR = Pulse rate, RBANS = Repeatable Battery for the Assessment of Neuropsychological Status, RR = Respiration rate.

## Data Availability

The data that support the findings of this study are available from the corresponding author, A.S., upon reasonable request.

## Supplementary Materials

The following are available online at https://www.mdpi.com/2077-0383/10/4/561/s1, Figure S1: Return to Activity Avoidance Inventory (RAAvI).
