# Supplementary material for: Cardiorespiratory Functioning in Youth with Persistent Post-Concussion Symptoms: A Pilot Study
Source: J Clin Med. 2021 Feb 3;10(4):561. doi: 10.3390/jcm10040561 (PMC7913264; doi:10.3390/jcm10040561)
Supplement: Supplementary file 1 [file jcm-10-00561-s001.pdf]

NAME____ DATE____________________ ID_____________________

UCLA Return to Activity Avoidance Inventory (RAAvI)

We'd like to know more about how your concussion has affected your ability to do the things you want to do. Some people who have had a concussion may avoid doing different activities because of symptoms (e.g., headaches, dizziness, anxiety, etc.) they experience since the concussion.

List the top three activities that you avoid (e.g., sports, homework, attending class/school, social gatherings). Please be as specific as possible. For example, instead of "sports" you may write "running":

Activity 1: ____

Activity 2: ____

Activity 3: ____

Think about **Activity 1** and answer the following questions:

1. How likely are you to avoid this activity?

None of the time	Some of the time	Half of the time	Most of the time	All of the time

2. How emotionally or physically distressed would you be doing this activity?

None	Mild	Moderate	Strong	Extreme

Now think about **Activity 2** and answer the following questions:

1. How likely are you to avoid this activity?

None of the time	Some of the time	Half of the time	Most of the time	All of the time

2. How emotionally or physically distressed would you be doing this activity?

None	Mild	Moderate	Strong	Extreme

Now think about **Activity 3** and answer the following questions:

1. How likely are you to avoid this activity?

None of the time	Some of the time	Half of the time	Most of the time	All of the time

2. How emotionally or physically distressed would you be doing this activity?

None	Mild	Moderate	Strong	Extreme
